# Factors associated with low-acuity hospital admissions in a public safety-net setting: a cross-sectional study

**DOI:** 10.1186/s12913-020-05456-3

**Published:** 2020-08-24

**Authors:** Noushyar Panahpour Eslami, Jefferson Nguyen, Luis Navarro, Madison Douglas, Maralyssa Bann

**Affiliations:** 1 University of Washington, Seattle, USA; 2 Division of GIM/Hospital Medicine, Harborview Medical Center, 325 9th Avenue, Box 359780, Seattle, WA 98104 USA; 3 Department of Medicine, University of Washington School of Medicine, Seattle, USA

**Keywords:** Hospital admission, Non medical acuity, Decision to admit

## Abstract

**Background:**

Given system-level focus on avoidance of unnecessary hospitalizations, better understanding admission decision-making is of utility. Our study sought to identify factors associated with hospital admission versus discharge from the Emergency Department (ED) for a population of patients who were assessed as having low medical acuity at time of decision.

**Methods:**

Using an institutional database, we identified ED admission requests received from March 1, 2018 to Feb 28, 2019 that were assessed by a physician at the time of request as potentially inappropriate based on lack of medical acuity. Focused chart review was performed to extract data related to patient demographics, socioeconomic information, measures of illness, and system-level factors such as previous healthcare utilization and day/time of presentation. A binary logistic regression model was constructed to correlate patient and system factors with disposition outcome of admission to the hospital versus discharge from the ED. Physician-reported contributors to admission decision-making and chief complaint/reason for admission were summarized.

**Results:**

A total of 349 (77.2%) of 452 calls resulted in admission to the hospital and 103 (22.8%) resulted in discharge from the ED. Predictors of admission included age over 65 (OR 3.5 [95%CI 1.1–11.6], *p* = 0.039), homelessness (OR 3.3 [95% CI 1.7–6.4], *p*=0.001), and night/weekend presentation (OR 2.0 [95%CI 1.1–3.5], *p* = 0.020). The most common contributing factors to the decision to admit reported by the responding physician included: lack of outpatient social support (35.8% of admissions), homelessness (33.0% of admissions), and substance use disorder (23.5% of admissions).

**Conclusions:**

Physician medical decision-making regarding the need for hospitalization incorporates consideration of individual patient characteristics, social setting, and system-level barriers. Interventions aimed at reducing unnecessary hospitalizations, especially those involving patients with low medical acuity, should focus on underlying unmet needs and involve a broad set of perspectives.

## Background

Inpatient hospitalizations account for nearly a third of the over $3 trillion spent on health care in the U.S. yearly [1]; reducing any unnecessary admissions is of great interest, though operationalizing this definition has been challenging. Hospitalization for an ambulatory care-sensitive condition is commonly considered “potentially preventable” based on the presumption that greater access to or higher quality of preventative care would avoid these hospital admissions [2–5]. The number of preventable (also called avoidable) admissions is widely used as a health care system quality indicator across the globe [6] and is an active target for cost containment strategies [7]. However, critiques of this approach point to a lack of validation studies, limited understanding of complex underlying contributors, and questions regarding whether these hospitalizations are truly preventable [8–12]. Some have argued the need for “[a] means of assessing preventability of individual admissions,” [13] though a tool developed for this purpose was subsequently found not to be valid [14].

Given the complexity surrounding assessment of preventability, a potentially informative related concept is identification of “inappropriate” admissions for which hospitalization is not thought to be necessary or of benefit to the patient. Functionally, this is often determined using medical records via utilization review [15] or by application of a standardized tool such as the Appropriateness Evaluation Protocol (AEP) [16]. The AEP is broadly used in the literature [17–23], though there have been some concerns regarding its reliability, validity, and methodologic application [24, 25]. More to the point, this approach retrospectively uses care delivered or objective findings as markers of appropriateness; it is generally unable to interrogate context at the time of admission decision and is not intended as a decisional tool for individual patient cases [26]. Thus, deeper analysis is warranted and further work “… to understand the process through which decisions about hospitalization are made in the ED” [27] has been identified as an important area of focus.

Hospital admission is ultimately a clinical decision made between physician and patient with multifaceted influences including system pressures, patient needs, and general practice culture [28–30]. Physicians report relying heavily on clinical gestalt over evidence-based protocols and often consider a holistic patient assessment, including so-called “extramedical” or “social” factors, [31–35] in place of or in addition to focused disease-specific evaluation. Better contextualization of this admission decision-making especially within a population of patients with low medical acuity at presentation would expand the current knowledge base by defining tangible targets for future interventions.

Few studies have captured clinician assessment of appropriateness based on medical acuity at time of admission, and to our knowledge, none have incorporated hospitalists who increasingly have defined roles as “triage physicians” in the decision to admit patients to acute care medical services [36]. Therefore, in order to better understand the circumstances of potentially unnecessary admissions, we aimed to explore admission versus discharge outcomes for a population of patients who presented to our safety-net hospital Emergency Department (ED) and were considered potentially inappropriate for hospitalization based on lack of medical acuity.

## Methods

### Study setting

This study was conducted at a 413-bed public county teaching hospital in Seattle, Washington that sees over 60,000 ED visits yearly. Our institution is the only level 1 trauma center for the surrounding region and serves a clinical mission to care for individuals unable to access or afford healthcare elsewhere.

At our site, the ED clinical team is responsible for providing stabilization, diagnosis, and referral to the appropriate potential inpatient service. All referral calls to the acute care medical service are answered by a Hospital Medicine attending physician (“triage physician”) who assesses need for hospitalization from the inpatient perspective and assists with any barriers to discharge. This triage physician may evaluate patients in-person if necessary or may rely on information relayed from the referring ED physician and from review of the medical record. Through this collaborative process, the triage physician arrives at a final admission decision for the patient or facilitates alternative disposition. During our study time period, a total of 36 Hospital Medicine physicians served as the triage physician.

### Triage database

At the end of each clinical shift the triage physician logs each call received into a central Triage Database including a response to the following question: “Based ONLY on the medical reason for hospitalization, in your opinion how appropriate is this admission to the Medicine floor service?” Available answer choices include “Definitely,” “Probably,” “Probably NOT,” or “Definitely NOT.” For any choice other than “Definitely,” an additional question offers the following selections: (1) “severity of medical problems alone may not require inpatient hospitalization;” (2) “better served on a different primary service;” (3) “meets ICU criteria/inappropriate or borderline for the floor.” A final question allows the triage physician to enter contributing factors considered in the ultimate admission decision from the following list: homelessness, substance use disorder, mental health disorder, physical limitation, cognitive limitation, low health literacy, non-English speaking, lack of outpatient social support, lack of outpatient medical support, or rejection from a skilled nursing facility or adult family home.

At the launch of the Triage Database, all triage physicians were oriented to the tool and instructed to log all calls received. In order to limit bias, no specific definition or criteria for “appropriateness for admission” were provided.

### Selection criteria

**Fig. 1:**
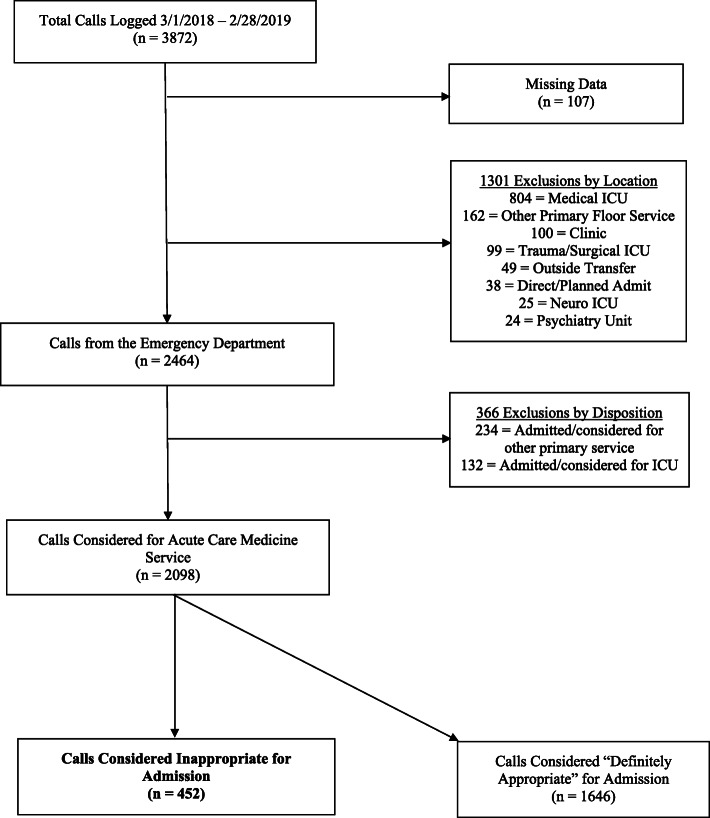
Call Selection Diagram

All calls for admission from the ED to the triage physician between March 1, 2018 and February 28, 2019 that were logged into the Triage Database were considered for this study. To be included, the triage physician must have assessed appropriateness as any category other than “Definitely” appropriate and selected the “severity of medical problems alone may not require inpatient hospitalization” follow-up response. Calls with insufficient Triage Database information were excluded. See Fig. 1 for selection flowchart. Out of a total 3872 calls logged in the Triage Database during the study time period, 452 met inclusion criteria.

### Outcome and variables of interest

Our primary outcome of interest was whether patients were admitted to the hospital or discharged from the ED. For this purpose, hospitalization required that an acute care medical team accepted responsibility for the patient via placement of an admission order and documentation of an admission note. If these markers were present, the outcome was coded as an admission. For any discrepancy, additional records such as ED documentation were reviewed to verify outcome. Admission status was not a definitional criterion; both inpatient and observation stays were considered admissions to the hospital.

For each case, a trained research assistant performed in-depth chart review to abstract relevant data. All information collected reflects what was reported in and available via the medical record. Results were reviewed in weekly research group meetings and any questions or discrepancies were addressed by the principal investigator.

Patient demographic information included age, gender (male vs. female), race/ethnicity (non-Hispanic White, Black/African American, Asian, American Indian/Alaska Native, Native Hawaiian/Pacific Islander, Hispanic/Latino, Multiple), English as primary language (yes/no), and marriage status (married vs. non-married, where non-married was defined as single, divorced, separated, or widowed). Socioeconomic factors included insurance (public, defined as Medicare, Medicaid, or both; private, defined as any commercial insurance product; or none), employment status (employed, unemployed, disabled, retired) and living situation (stable housing, defined as independent living with a permanent address on file; unstable housing/homeless, defined by social worker documentation and/or as no address or shelter address on file; institutional housing, defined as skilled nursing facility, adult family home, assisted living facility, or jail/prison). Measures of illness included age-adjusted Charlson Comorbidity Index (CCI) score and the Emergency Severity Index (ESI) score [37] assigned by ED providers at time of arrival. System-level factors included off-hours presentation (defined as time of ED registration on weekdays between 5 PM and 7 AM or anytime Saturday or Sunday) and number of visits to any ED in the previous 30 days (as reported through a shared regional data tool within the medical record). The chief complaint entered by the triage physician was categorized into four themes: “diagnosis/syndrome”, “undifferentiated symptoms”, “abnormal objective laboratory or vital sign measurement”, and “social disposition”.

### Statistical analysis

Summary statistics and comparisons were calculated between disposition groups (admission to the hospital vs. discharge from ED). Any missing data element is denoted in the corresponding tables and was removed from calculation. Categorical data were compared using a χ2 test and continuous data were compared by Student’s t-test. Continuous variables are presented as means with 95% confidence intervals (CI). A binary multivariable logistic regression model was constructed using statistically significant univariable results. Hosmer-Lemeshow statistics were calculated for model fitness. Results are reported as odds ratios (ORs) with 95% confidence intervals using a 2-sided significance threshold of *P* <  0.05. For model purposes, age was categorized into clinically relevant groupings (18–30; 31–50; 51–65; > 65 years). To assess for potential confounding, variable comparisons by appropriateness category were conducted and no statistical differences were identified (see Supplemental Table 1). Statistical analysis was conducted using STATA (version 15.1, College Station, TX).

All study data were collected and managed using REDCap electronic data capture tools hosted at University of Washington [38, 39]. The study was approved by the Institutional Review Board at the University of Washington. This manuscript adheres to the EQUATOR STROBE Checklist for cross-sectional studies [40].

## Results

### Appropriateness assessment

Of the total 452 calls included, 253 (56.0%) were assessed as “Probably” appropriate; 158 (35.0%) as “Probably NOT” appropriate; and 41 (9.0%) as “Definitely NOT” appropriate. We did not find any differences between appropriateness categories with respect to patient demographics, socioeconomic factors, or measures of illness (Supplemental Table 1).

### Patient demographics, socioeconomic factors, and measures of illness

Patients in this study were middle-aged (mean 55.8 years; 95% CI 54.3–57.3), predominantly male (286/452; 63.3%), English-speaking (371/452; 82.1%), and covered by public insurance (388/452; 85.8%). Roughly half were non-Hispanic white (218/448; 48.7%). Relatively few patients reported being married (78/452; 17.3%) or employed (26/429; 6.1%), while a large portion experienced homelessness prior to admission (148/449; 33.0%). For included patients, the mean CCI score was 3.23 (95%CI 2.98–3.49) and the mean ESI score was 2.84 (95%CI 2.79–2.89). Summary statistics are presented in Table 1.

**Table 1:** Comparison of Admission vs. Discharge from ED by Patient Demographics, Socioeconomic Factors, Measures of Illness, System Factors, and Appropriateness of Admission

	All Patients (*n* = 452)	Admitted from ED (*n* = 349)	Discharged from ED (*n* = 103)	P
Patient Demographics				
Age, mean [95%CI]	55.8 [54.3–57.3]	56.6 [54.9–58.3]	53.0 [49.7–56.2]	0.046
Female, n (%)	166 (36.7)	132 (37.8)	34 (33.0)	0.373
Race/Ethnicity, n (%), 4 missing				
American Indian/Alaska Native	22 (4.9)	17 (4.9)	5 (4.9)	0.876
Asian	39 (8.7)	31 (9.0)	8 (7.8)	
Black/African-American	122 (27.2)	90 (26.0)	32 (31.4)	
Hispanic/Latino	36 (8.0)	26 (7.5)	10 (9.8)	
Hawaiian/Pacific Islander	4 (0.9)	3 (0.9)	1 (1.0)	
White, non-Hispanic	218 (48.7)	174 (50.3)	44 (43.1)	
Multiple	7 (1.6)	5 (1.4)	2 (2.0)	
English Primary Language, n (%)	371 (82.1)	291 (83.4)	80 (77.7)	0.184
Married, n (%)	78 (17.3)	56 (16.0)	22 (21.4)	0.210
Socioeconomic Factors				
Insurance, n (%)				0.058
Public	388 (85.8)	307 (90.1)	81 (78.6)	
Private	21 (4.7)	14 (4.0)	7 (6.8)	
None	43 (9.5)	28 (8.0)	15 (14.6)	
Employment, n (%), 23 missing				0.397
Employed	26 (6.1)	18 (5.3)	8 (8.8)	
Unemployed	161 (37.5)	123 (36.4)	38 (41.8)	
Disabled	112 (26.1)	91 (26.9)	21 (23.1)	
Retired	130 (30.3)	106 (31.4)	24 (26.4)	
Living Situation, n (%), 3 missing				0.008
Stable housing	261 (58.1)	190 (54.9)	71 (69.0)	
Unstable housing/homeless	148 (33.0)	127 (36.7)	21 (20.4)	
Institution (SNF/AFH/Jail)	40 (8.9)	29 (8.4)	11 (10.7)	
Measures of Illness				
CCI, mean [95%CI]	3.23 [2.98–3.49]	3.31 [3.02–3.60]	2.96 [2.40–3.52]	0.265
ESI, mean [95%CI]	2.84 [2.79–2.89]	2.87 [2.81–2.92]	2.75 [2.64–2.85]	0.041
System-Level Factors				
“Off-hours” presentation, n (%)	287 (63.5)	232 (66.5)	55 (53.4)	0.015
Healthcare Utilization				
ED visits in previous 30 days, n (%)				0.652
None	265 (58.6)	207 (59.3)	58 (56.3)	
1 visit	103 (22.8)	82 (23.5)	21 (20.4)	
2 visits	46 (10.2)	32 (9.2)	14 (13.6)	
3–5 visits	32 (7.1)	24 (6.9)	8 (7.8)	
6+ visits	6 (1.3)	4 (1.1)	2 (2.0)	
Appropriateness Assessment, n (%)				< 0.0001
Probably	253 (56.0)	246 (70.5)	7 (6.8)	
Probably NOT	158 (34.9)	80 (22.9)	78 (75.7)	
Definitely NOT	41 (9.1)	23 (6.6)	18 (17.5)	

*CCI* Charlson Comorbidity Index, *ESI* Emergency Severity Index, *SNF* Skilled Nursing Facility, *AFH* Adult Family Home

### Chief complaint or reason for admission

Chief complaint or reason for admission as described by the triage physician is summarized in Table 2. As compared to those discharged from the ED, patients admitted to the hospital more often had a defined diagnosis or syndrome (56.2% vs 49.5%) or presented requiring a need for social “placement” (7.2% vs. 0%). Patients discharged from the ED more often presented with undifferentiated symptoms (35.9% vs. 29.5%) or abnormal objective measurements such as laboratory values or vital signs (14.6% vs. 4.6%).

**Table 2:** Chief Complaint or Reason for Admission per Triage Physician

	Admitted from ED (*n* = 349)	Discharged from ED (*n* = 103)
Diagnosis/Syndrome, n (%)	196 (56.2)	51 (49.5)
	Skin/soft tissue infection [40]	Skin/soft tissue infection [10]
	ESRD/hemodialysis [13]	Intoxication/withdrawal [5]
	COPD [13]	ESRD/hemodialysis [4]
Undifferentiated Symptom, n (%)	103 (29.5)	37 (35.9)
	Ambulatory dysfunction [26]	Pain [16]
	Pain [22]	Ambulatory dysfunction [6]
	Altered mental status [17]	Dyspnea/cough [5]
Objective Laboratory or Vital Sign Measurement, n (%)	16 (4.6)	15 (14.6)
	Anemia [2]	Anemia [5]
	Hyponatremia [2]	Hyperkalemia [2]
	Hypoxia [2]	Hypotension [2]
Social “Placement”, n (%)	25 (7.2)	0
Missing/not recorded, n (%)	9 (2.6)	0

The three most common presenting complaints for each category are listed with frequency [*n*] ESRD=end-stage renal disease, COPD= chronic obstructive pulmonary disease

Among the most common diagnoses or syndromes, skin/soft tissue infection and end stage renal disease (ESRD)/hemodialysis were reported in both admitted and discharged groups with chronic obstructive pulmonary disease (COPD) noted in the admitted group and intoxication/withdrawal syndromes in the discharged group. Ambulatory dysfunction and pain complaints were common undifferentiated symptoms in both groups along with altered mental status reported in the admitted group and dyspnea/cough in the discharged group. Finally, of abnormal objective measurements, anemia was common in both groups followed by hyponatremia and hypoxia in the admitted group and hyperkalemia and hypotension in the discharged group.

### Comparison of admission vs. discharge groups

A total of 349 (77.2%) of the 452 calls resulted in admission to the hospital and 103 (22.8%) resulted in discharge from the ED. Comparisons between these groups are summarized in Table 1. Admitted patients were older than those discharged from the ED (mean age 56.6 years [95% CI 54.9–58.3] vs. 53.0 years [95%CI 49.7–56.2], *p* = 0.046). There were no differences by patient gender, race/ethnicity, primary English-speaking, or marriage status. Differences in living situation were noted with a higher proportion of patients experiencing unstable housing or homelessness in the group admitted to the hospital (36.7% vs. 20.4%, *p*=0.008), but no differences were noted by insurance or employment status. While there was no significant difference regarding the CCI scores (admitted mean score 3.31 [95%CI 2.76–3.02] vs. discharged mean score 2.96 [95%CI 2.40–3.52], *p* = 0.265), those who were admitted had a higher (less acute) ESI score than those discharged (mean score 2.87 [95%CI 2.79–2.89] vs. 2.75 [95%CI 2.64–2.85], *p* = 0.041). More patients within the admitted group presented to the ED during “off-hours” (66.5% vs. 53.4%, *p* = 0.015). There was no difference between groups in ED visits within the previous 30 days. Appropriateness assessment categorization differed between groups with a large proportion of admitted patients deemed “Probably” appropriate as compared to discharged patients (70.5% vs. 6.8%), *p* <  0.0001).

### Factors associated with admission

In the binary logistic regression model, predictors of admission included age over 65 (OR 3.5 [95%CI 1.1–11.6], *p* = 0.039), homelessness (OR 3.3 [95% CI 1.7–6.4], *p* = 0.001), night/weekend presentation (OR 2.0 [95%CI 1.1–3.5], *p* = 0.020), and admission appropriateness category (“Probably NOT”: OR 0.02 [95%CI 0.01–0.06], *p <*  0.0001; “Definitely NOT”: OR 0.03 [95%CI 0.01–0.09], *p <*  0.0001). ESI score was not statistically significant in the regression model. These results are presented in Table 3.

**Table 3:** Factors Associated with Admission in Logistic Regression Analysis

	Odds Ratio (95%CI)	P
Patient Age		
18–30	Reference	
31–50	1.0 (0.3–3.2)	0.96
51–65	1.7 (0.5–5.5)	0.37
> 65	3.5 (1.1–11.6)	0.039
Living Situation		
Stable housing	Reference	
Unstable housing/homeless	3.3 (1.7–6.4)	0.001
Institution (SNF/AFH/Jail)	1.3 (0.5–3.2)	0.59
“Off-hours” presentation	2.0 (1.1–3.5)	0.020
Appropriateness		
Probably	Reference	
Probably NOT	0.02 (0.01–0.06)	< 0.0001
Definitely NOT	0.03 (0.01–0.09)	< 0.0001

Number of obs 449, LRchi2 = 172.27, *p* = 0.0000, pseudoR2 = 0.3562

### Contributors to admission per triage physician

Contributors to admission as reported by the triage physician are summarized in Table 4. Overall, among patients who were admitted to the hospital, the most common factors that triage physicians reported contributing to the decision were: lack of outpatient social support (35.8%), homelessness (33.0%), and substance use disorder (23.5%). This pattern was similar for the subgroup of patients who presented during “off-hours” (lack of outpatient social support 36.2%, homelessness 31.9%, substance use disorder 24.1%). For patients experiencing unstable housing/homelessness, though the three most common contributors remained the same, the order and prevalence differed (homelessness 80.3%, lack of outpatient social support 40.9%, substance use disorder 37.0%). For patients over age 65, two of the top three contributors differed (lack of outpatient social support 42.1%, cognitive limitation 25.2%, physical limitation 23.4%).

**Table 4:** Triage Physician Reported Contributors to Admissions

	All Admissions (*n* = 349)	Admissions by Associated Factor		
		Age Over 65 (*n* = 107)	Unstable Housing/ Homeless (*n* = 127)	“Off-Hours” Presentation (*n* = 232)
Lack of Outpatient Social Support (Family, Caregiver, etc.)	**125 (35.8)**	**45 (42.1)**	**52 (40.9)**	**84 (36.2)**
Homelessness	**115 (33.0)**	16 (15.0)	**102 (80.3)**	**74 (31.9)**
Substance Use Disorder	**82 (23.5)**	14 (13.1)	**47 (37.0)**	**56 (24.1)**
Lack of Outpatient Medical Support	81 (23.2)	19 (17.8)	34 (26.8)	53 (22.8)
Physical Limitation	69 (19.8)	**25 (23.4)**	26 (20.5)	46 (19.8)
Mental Health Disorder	63 (18.1)	12 (11.2)	31 (24.4)	40 (17.2)
Cognitive Limitation	50 (14.3)	**27 (25.2)**	18 (14.2)	33 (14.2)
Low Health Literacy	39 (11.2)	11 (10.3)	16 (12.6)	23 (9.9)
Non-English Speaker	22 (6.3)	9 (8.4)	3 (2.4)	11 (4.7)
SNF or Adult Family Home Refusal	18 (5.2)	9 (8.4)	4 (3.1)	13 (5.6)

**Most common contributors bolded for each category**

Note: Multiple selections were allowed, so percentages do not equal 100%

## Discussion

This study takes the approach of selecting cases for inclusion based upon lack of appropriateness for admission due to low medical acuity as assessed by a physician at the time of admission. Using this method incorporates an individualized assessment that is distinct from the concept of “preventable” ambulatory care sensitive condition admission and avoids reliance on retrospective “inappropriate” admission scoring tools. While recognizing that patient care requires a collaborative and interprofessional approach, we specifically focus on the triage physician decisions since both the decision to admit and admission appropriateness assessment are determined by the same individual. This allows investigation of the following questions: 1) How frequently does a physician admit a patient to the hospital despite also determining that the patient lacks sufficient medical acuity to warrant hospitalization and 2) What contributes to this decision?

We found that over three-quarters of patients considered not acutely ill enough to warrant hospitalization were ultimately admitted to an acute care medical service. The admitted group had a higher (less acute) average ESI score than the discharged group, further corroborating that factors outside of presenting medical acuity contribute to ultimate disposition. Overall, our findings appear consistent with previous report that a significant proportion of hospitalizations from the ED may be affected by issues other than medical acuity [35]. In our study, advanced age, homelessness, and night/weekend “off-hours” presentation were each associated with increased odds of admission and physicians reported that issues such as lack of family/caregiver commonly impacted their admission decisions.

Taken together, this suggests that acute care hospitalization plays an important role in the context of unmet social support needs and that physicians consider these needs in admission decision-making. Furthermore, we have reported patterns of physician considerations that suggest a more nuanced view of unnecessary admissions. For example, frailty is a known predictor of hospitalization for geriatric populations [41, 42] and here we describe that physicians specifically report concern for physical and/or cognitive limitations as part of their decisions to admit older patients without medical acuity. Likewise, previous research indicates that individuals experiencing homelessness are more likely to be hospitalized [43] and that substance use is a significant cause of mortality in this population [44]. This is consistent with our findings that homelessness and substance use disorder are major sources of concern for physicians in the admission decision, especially for patients presenting during off-hours.

Health system interventions aimed at providing additional levels of patient support such as care coordination efforts [45], intensive primary care strategies [46], health education initiatives [47], and community health worker programs [48] have shown inconsistent reduction in hospitalizations. Even Medicaid expansion, despite increasing access and coverage, seems primarily to have changed the payer but not the number of admissions [49–51]. This is perhaps unsurprising given the concerns that underlie physician decision-making identified in this study. Ultimately, the response to reducing unnecessary hospitalizations will likely require interventions that are broader than a healthcare-based response. For example, programs that provide stable housing show promise in this regard [52–54] as do substance use disorder treatment and comprehensive support efforts [55]. These types of approaches warrant additional consideration.

Our findings should be considered in context given that this study was conducted at a single site safety-net institution. Availability of resources, population needs, and general practice may vary. For example, our site does not have a dedicated ED observation unit, so we may have captured cases in this study that would be handled differently elsewhere. We restricted our study population to patients considered for admission to an acute care medical ward, which at our site does not include acute cardiac or neurologic patients, though this might be true in sites where a broader patient population is admitted by generalists. Finally, physician assessment is inherently subjective and using it to determine included cases may introduce variability or bias. However, if anything, one would hypothesize that physicians would report more concurrence between their own appropriateness assessment and ultimate disposition decision, which is inconsistent with the high proportion of patients admitted despite assessment of low medical acuity. Since admission decision-making was the focus of our inquiry and there is no robust scoring tool that is able to capture the nuances of clinician judgment, we argue that this is an appropriate approach.

## Conclusions

A large proportion of patients assessed as lacking definite medical acuity that would warrant hospitalization was ultimately admitted to the acute care medical service. Older age, homelessness/unstable housing, and night/weekend presentation were each associated with increased odds of admission. This appears to be related to physician medical decision-making that incorporates consideration of individual patient characteristics, social setting, and system-level barriers. Future work to decrease unnecessary admissions will need to better understand this context as well as the perceived role of the hospital within the social safety net.

## Supplementary information

**Additional file 1: Supplemental Table 1**. Patient Demographics and Measures of Illness by Appropriateness Assessment

## Data Availability

The datasets generated and/or analyzed during the current study are not publicly available for protection of potentially identifiable information.

## Abbreviations

ED: Emergency Department
AEP: Appropriateness Evaluation Protocol
CCI: Charlson Comorbidity Index
ESI: Emergency Severity Index
CI: Confidence Interval
