# Supplementary material for: Factors associated with low-acuity hospital admissions in a public safety-net setting: a cross-sectional study
Source: BMC Health Serv Res. 2020 Aug 24;20:775. doi: 10.1186/s12913-020-05456-3 (PMC7446119; doi:10.1186/s12913-020-05456-3)
Supplement: Supplementary file 1 — Additional file 1: Supplemental Table 1. Patient Demographics and Measures of Illness by Appropriateness Assessment [file 12913_2020_5456_MOESM1_ESM.docx]

**Supplement**

Supplemental Table 1. Patient Demographics and Measures of Illness by Appropriateness Assessment

CCI = Charlson Comorbidity Index; ESI = Emergency Severity Index; SNF = Skilled Nursing Facility; AFH = Adult Family Home

|  | Probably Appropriate  N = 253 | Probably NOT  Appropriate  N = 158 | Definitely NOT Appropriate  N = 41 |  |
| --- | --- | --- | --- | --- |
| Age, mean [95%CI] | 55.5 [53.5-57.5] | 55.9 [53.4-58.4] | 57.0 [51.9-62.0] | 0.864 |
| Female, n (%) | 92 (36.4) | 60 (38.0) | 14 (34.1) | 0.888 |
| Race/Ethnicity, n (%), *4 missing* |  |  |  | 0.116 |
| AI/AN | 13 (5.2) | 9 (5.7) | 0 (0) |  |
| Asian | 28 (11.2) | 10 (6.4) | 1 (2.5) |  |
| Black/AA | 57 (22.7) | 49 (31.2) | 16 (40.0) |  |
| Hispanic/Latino | 20 (8.0) | 14 (8.9) | 2 (5.0) |  |
| Hawaiian/PI | 3 (1.2) | 1 (0.6) | 0 (0) |  |
| White, non-Hispanic | 128 (50.1) | 71 (45.2) | 19 (47.5) |  |
| Multiple | 2 (0.8) | 3(1.9) | 2 (5.0) |  |
| English Primary Language, n (%) | 210 (83.0) | 125 (79.1) | 36 (87.8) | 0.367 |
| Married, n (%) | 37 (14.6) | 30 (19.0) | 11 (26.8) | 0.123 |
| Insurance, n (%) |  |  |  | 0.750 |
| Public | 221 (87.4) | 133 (84.2) | 34 (82.9) |  |
| Private | 9 (3.6) | 9 (5.7) | 3 (7.3) |  |
| None | 23 (9.1) | 16 (10.1) | 4 (9.8) |  |
| Employment, n (%), *23 missing* |  |  |  | 0.186 |
| Employed | 12 (4.9) | 12 (8.0) | 2 (5.7) |  |
| Unemployed | 99 (40.6) | 53 (35.3) | 9 (25.7) |  |
| Disabled | 67 (27.5) | 32 (21.3) | 13 (37.1) |  |
| Retired | 66 (27.0) | 53 (35.3) | 11 (31.4) |  |
| Living Situation, n (%), *3 missing* |  |  |  | 0.363 |
| Stable Housing | 143 (57.0) | 94 (59.9) | 24 (58.5) |  |
| Institutionalized | 18 (7.2) | 16 (10.2) | 6 (14.6) |  |
| Homeless | 90 (35.9) | 47 (30.0) | 11 (26.8) |  |
| CCI, mean [95%CI] | 3.14 [2.81-3.48] | 3.35 [2.89-3.82] | 3.29 [2.51-4.07] | 0.337 |
| ESI, mean [95%CI] | 2.87 [2.80-2.93] | 2.78 [2.69-2.86] | 2.90 [2.71-3.09] | 0.237 |
| ED Presentation in previous 30 days |  |  |  | 0.850 |
| None | 156 (61.7) | 87 (55.1) | 22 (53.7) |  |
| 1 visit | 53 (20.9) | 38 (24.1) | 12 (29.3) |  |
| 2 visits | 24 (9.5) | 17 (10.8) | 5 (12.2) |  |
| 3-5 visits | 17 (6.7) | 13 (8.2) | 2 (4.9) |  |
| 6+ visits | 3 (1.2) | 3 (1.9) | 0 (0) |  |
